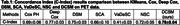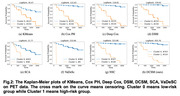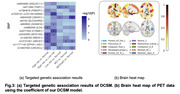# Interpretable Deep Clustering Survival Machines for Alzheimer's Disease Risk Groups Discovery

**DOI:** 10.1002/alz.092319

**Published:** 2025-01-09

**Authors:** Bojian Hou, Zixuan Wen, Jingxuan Bao, Richard Zhang, Boning Tong, Shu Yang, Junhao Wen, Yuhan Cui, Jason H. Moore, Andrew J. Saykin, Heng Huang, Paul M. Thompson, Marylyn D. Ritchie, Christos Davatzikos, Li Shen, Alzheimer's Disease Neuroimaging Initiative

**Affiliations:** ^1^ University of Pennsylvania, Philadelphia, PA USA; ^2^ University of Southern California, Los Angeles, CA USA; ^3^ Cedars‐Sinai Medical Center, West Hollywood, CA USA; ^4^ Indiana University, Indianapolis, IN USA; ^5^ University of Maryland, College Park, MD USA

## Abstract

**Background:**

Alzheimer's disease (AD) is a complex neurodegenerative disorder that has impacted millions of people worldwide. Identifying different risk groups converting to AD during the mild cognitive impairment (MCI) stage and determining their genetic basis would be immensely valuable for drug discovery and subsequent clinical treatment. Previous studies typically clustered subgroups by unsupervised learning techniques, neglecting the survival information. To address this problem, we propose an interpretable survival analysis method called Deep Clustering Survival Machines (DCSM), which performs clustering and risk prediction simultaneously.

**Method:**

Our proposed DCSM is a hybrid survival analysis method that integrates the advantages of the discriminative and generative ideas and employ the mixture of *expert distributions* to fit the final survival function by maximum likelihood estimation. For the real data analysis, we use positron emission tomography (PET) from the ADNI dataset as feature. The survival information includes whether the patients convert from MCI to AD and the corresponding time. Our DCSM model predicts the risk of each patient and clusters the patients into two groups based on their risk simultaneously.

**Result:**

We first evaluate time‐to‐event prediction performance by Concordance Index which is shown in Table 1 where our model outperforms the other baselines. Besides, we use the LogRank to evaluate the clustering performance. The difference in survival between two risk groups as shown in Kaplan‐Meier plots in Figure 2(h) is significantly larger than other baselines. Furthermore, to biologically validate our clustering findings, we carry out targeted genetic association analyses (Figure 3(a)). There are numerous targeted SNPs discovered when comparing high‐risk group to low‐risk group and normal group. Figure 3(b) illustrates the important brain regions for the high‐risk group. We highlight the top ten important regions which is consistent with the conventional brain‐AD relation findings.

**Conclusion:**

Our DCSM model builds a good bridge between the imaging modality and survival information. We demonstrate the superiority of the DCSM by applying this approach to cluster patients with MCI into subgroups with different risks of converting to AD. Conventional clustering measurements for survival analysis along with genetic association studies successfully validate the effectiveness of our method and characterize our clustering findings.